# Macrophage polarization in peri-implantitis lesions

**DOI:** 10.1007/s00784-020-03556-2

**Published:** 2020-09-04

**Authors:** Maria Elisa Galarraga-Vinueza, Karina Obreja, Ausra Ramanauskaite, Ricardo Magini, Amira Begic, Robert Sader, Frank Schwarz

**Affiliations:** 1 Department of Oral Surgery and Implantology, Johann Wolfgang Goethe-University, Carolinum, Frankfurt, Germany; 2 Post-Graduate Program in Implant Dentistry (PPGO), Federal University of Santa Catarina (UFSC), Florianopolis, SC Brazil; 3 School of Dentistry, Universidad de Las Américas, Quito, Ecuador; 4 Department for Oral, Cranio-Maxillofacial and Facial Plastic Surgery, Medical Center of the Goethe University Frankfurt, Frankfurt am Main, Germany; 5 Department of Oral Surgery, Universitätsklinikum Düsseldorf, Dusseldorf, Germany

**Keywords:** Biopsy, Dental implant, Immunohistochemistry, Peri-implantitis, Macrophage polarization

## Abstract

**Objectives:**

To immunohistochemically characterize and correlate macrophage M1/M2 polarization status with disease severity at peri-implantitis sites.

**Materials and methods:**

A total of twenty patients (*n* = 20 implants) diagnosed with peri-implantitis (i.e., bleeding on probing with or without suppuration, probing depths ≥ 6 mm, and radiographic marginal bone loss ≥ 3 mm) were included. The severity of peri-implantitis was classified according to established criteria (i.e., slight, moderate, and advanced). Granulation tissue biopsies were obtained during surgical therapy and prepared for immunohistological assessment and macrophage polarization characterization. Macrophages, M1, and M2 phenotypes were identified through immunohistochemical markers (i.e., CD68, CD80, and CD206) and quantified through histomorphometrical analyses.

**Results:**

Macrophages exhibiting a positive CD68 expression occupied a mean proportion of 14.36% (95% CI 11.4–17.2) of the inflammatory connective tissue (ICT) area. Positive M1 (CD80) and M2 (CD206) macrophages occupied a mean value of 7.07% (95% CI 5.9–9.4) and 5.22% (95% CI 3.8–6.6) of the ICT, respectively. The mean M1/M2 ratio was 1.56 (95% CI 1–12–1.9). Advanced peri-implantitis cases expressed a significantly higher M1 (%) when compared with M2 (%) expression. There was a significant correlation between CD68 (%) and M1 (%) expression and probing depth (PD) values.

**Conclusion:**

The present immunohistochemical analysis suggests that macrophages constitute a considerable proportion of the inflammatory cellular composition at peri-implantitis sites, revealing a significant higher expression for M1 inflammatory phenotype at advanced peri-implantitis sites, which could possibly play a critical role in disease progression.

**Clinical relevance:**

Macrophages have critical functions to establish homeostasis and disease. Bacteria might induce oral dysbiosis unbalancing the host’s immunological response and triggering inflammation around dental implants. M1/M2 status could possibly reveal peri-implantitis’ underlying pathogenesis.

## Introduction

Contemporary evidence has pointed macrophages as central players in immune-inflammatory processes [1, 2]. Currently, macrophage’s function and phenotype polarization are major fields of research aiming to comprehend the inflammatory conditions that lead to diseases such as arteriosclerosis, diabetes type II, obesity, and periodontitis [3–5]. In fact, macrophages have essential functions to establish homeostasis and disease due to their phagocytic capacity and high cellular plasticity. In particular, certain mechanisms like epigenetic deregulations, disruption of the periodontium/peri-implant tissue homeostasis, and a dysfunctional immunological response to bacterial endotoxins can trigger inflammatory responses at tooth and implant sites [6, 7]. The initiation or resolution of inflammation might be governed by macrophage phenotype alternation which is dependent on different environmental signals [8, 9]. Accordingly, macrophages activated by bacteria sub-products (i.e., lipopolysaccharides) or interferon (IFN)-g present a M1 phenotype and are associated with pro-inflammatory responses, phagocytosis, tissue destruction, and interleukin (IL)-6 and IL-1β production. In contrast, alternative macrophages revealing a M2 phenotype are associated with anti-inflammatory reactions, including the production of IL-10 and transforming growth factor (TGF)-β to induce tissue repair and angiogenesis [1, 2, 10].

Peri-implantitis has been characterized as a plaque associated inflammatory pathological condition occurring in tissues around dental implants [11–14]. A few histological studies have reported on the cellular composition at peri-implantitis sites in humans [15, 16]. Carcuac and Berglundh reported in a histopathological analysis of tissue biopsies that peri-implantitis lesions contained large proportions of inflammatory cells and that specifically macrophages occupied 11% of the inflammatory cell composition. Nevertheless, macrophage’s phenotype expression and its role on the progression of peri-implant disease are not completely understood. So far, there are limited studies assessing macrophage polarization in periodontal disease which suggest that the pathogenesis of periodontitis could be related to an enhanced M1 inflammatory expression [4, 10, 17]. In particular, Zhou et al. reported that M1 expression was higher in human periodontitis biopsies when compared with gingivitis and healthy specimens. The latter study revealed a superior M1/M2 ratio accompanied with a higher expression of inflammatory cytokines (i.e., IL-6, IL-12, and TNF-alfa) at periodontitis sites. Divergently, Garaicoa-Pazmiño et al. reported a higher macrophage infiltration at gingivitis and periodontitis sites, however, an unexpected higher M2 expression at the mentioned diseased sites when compared with healthy tissues. Currently, there is solely one study comparing macrophage polarization status at human periodontitis and peri-implantitis lesions. The mentioned study exhibited that peri-implantitis sites revealed a higher macrophage M1 expression when compared with periodontitis sites [7].

Bearing in mind that there is scarce evidence on macrophage M1/M2 polarization role in peri-implant disease, a further assessment of macrophage polarization at peri-implantitis sites may help to better understand the underlying pathogenesis. Consequently, the present analysis aimed at immunohistochemically characterize and correlate macrophage M1/M2 polarization status with disease severity at peri-implantitis sites.

## Materials and methods

### Study design

In the present analysis, a total of 20 patients (*n* = 20 implants, mean age: 66.7 years; range: 53 to 80 years), who attended the Department of Oral Surgery and Implantology, Goethe University, Frankfurt, Germany for surgical treatment of peri-implantitis, were included (period of recruitment: May–November 2019). Peri-implantitis was defined as the combination of bleeding on gentle probing (BOP) with/without suppuration, probing depths (PD) ≥ 6 mm, and radiographic marginal bone loss (MBL) (i.e., “interproximal bone levels ≥ 3 mm apical of the most coronal portion of the intraosseous part of the implant”) [18].

The severity of peri-implantitis was classified according to established criteria (i.e., slight, moderate, and advanced) considering the defect length from the implant neck and ratio of MBL relative to the total implant length (bone loss %). As follows, slight, moderate, and advanced grades were determined if the MBL was < 25%, ≥ 25–50%, and > 50% of the implant length, respectively [19].

The study protocol no. 92/19 was approved by the ethics committee of Goethe-University, Frankfurt-Germany, 2019, and considered the Helsinki Declaration of 1975, as revised in 2013. Each patient was provided with detailed information of the study protocol and was required to sign an informed consent form.

### Selection and enrollment of participants

#### Inclusion criteria

For patient selection, the following inclusion criteria were considered: (1) patients who signed and approved the consent form, (2) minimum age of 18 years old, (3) partially/totally edentulous patients rehabilitated with a single/multiple implant-supported prosthesis, (4) the presence of at least one screw-type (one- or two-part) titanium implant diagnosed with peri-implantitis and indicated for surgical peri-implantitis treatment, (5) no implant mobility, (6) adequate oral hygiene as evidenced by a plaque index < 1 [20], and (7) non-smokers.

#### Exclusion criteria

The exclusion criteria considered patients who presented (1) general contraindications for dental and surgical treatments, (2) untreated periodontal disease, (3) pregnant or lactant women, (4) autoimmune or/and inflammatory diseases, (5) uncontrolled diabetes (HbA1c > 7), (6) corticosteroid therapy, and (7) smokers.

### Clinical and radiological examination

The following clinical parameters were assessed at each implant site using a periodontal probe (PCV12PT Hu-Friedy Inc., Chicago, IL, USA): (1) keratinized mucosa (KM) width; (2) BOP, evaluated as present if bleeding was evident within 30 s after probing, or absent, if no bleeding was noticed within 30 s after probing; (3) PD as measured from the mucosal margin to the bottom of the probable pocket; and (4) plaque index (PI) [20], mucosal recession (MR) as measured from the mucosal margin to the crown margin, and suppuration, evaluated as present if evident after probing and/or peri-implant palpation. All measurements were performed at 6 aspects per implant: mesio-vestibular, mid-vestibular, disto-vestibular, mesio-oral, mid-oral, and disto-oral by a calibrated investigator (K.O). MBL assessments were accomplished through radiological evaluation using a software program (Image J, Wisconsin, USA). MBL linear measurements were performed by drawing a vertical line, following the long axis of the implant, from the implant shoulder to the bottom of the defect at distal and mesial sites. The measurement scale was set by means of the known implant length. Radiological evaluations were performed by one experienced and calibrated examiner (M.E.G.).

Previous to the radiological analysis, an intra-examiner calibration was implemented to assess the reproducibility and consistency of the measurements taken by the mentioned methodology. The calibration was accepted when repeated measurements (*n* = 10) presented a intraclass correlation coefficient (ICC) ranging from 0.81 to 1.

### Sample collection and histological processing

All patients received pre-operative professional supra-gingival tooth/implant cleaning and were treated through a standardized surgical protocol described in detail previously [21]. In brief, following local anesthesia (articaine, 1:200.000), buccal and lingual mucoperiostal flaps were elevated to expose the peri-implant defect. All granulation tissue was carefully and circumferentially harvested from the respective intrabony defect area using conventional plastic curettes (Straumann Dental Implant System; Institut Straumann AG, Basel, Switzerland), rinsed with saline, and placed in 4% buffered formalin for 24 h. The biopsies were stored in 70% ethanol at 4 °C, dehydrated, and embedded in paraffin. Serial sections (4 μm thick) were cut and mounted on glass poly-D-lysine coated slides.

### Immunohistochemistry

Paraffin-embedded sections were dewaxed in xylene, rehydrated through graded alcohol, and incubated in antigen retrieval solution (ethylenediaminetetraacetic, EDTA) for 20 min. The histological sections were then incubated in 3% bovine serum albumin (BSA) diluted in phosphate buffer solution (PBS) for blocking procedure. Next, sections were incubated with primary antibodies for 40 min at 37 °C. Primary mouse monoclonal antibody against CD68 (1:200, NCL-L, Abcam, USA), rabbit monoclonal antibody against CD80 (1:200, EPR11572, Abcam, USA) and rabbit polyclonal mannose receptor antibody against CD206 (1:300, AB64693, Abcam, USA) were used to identify macrophages, M1 and M2 phenotypes, respectively. Sections were washed with PBS and incubated with a labeled polymer for 30 min and then with a substrate/chromogen for 10 min (EnVision Detection, DAKO, Denmark). Counterstaining was executed with Mayer’s hematoxylin. Negative controls were performed by replacing the primary antibody with non-immune serum.

### Histological analysis

Immunostained histological sections were scanned (× 40 magnification) with a Nikon E200 microscope using a digital virtual light microscopy system. The histological quantitative evaluation was performed using a software image system (Nikon, NIS, Basic Research, Japan) by analyzing the entire section of each specimen. First, the surface area (μm^2^) of the infiltrated connective tissue (ICT) was delimited by outlining its perimeter with a digital pen. The selected area was divided with a digital grid containing squares of 100 μm^2^. The area covered by each square was observed at a magnification of × 40. The stained areas (brown pigmented) depicting positive cells for each immunohistochemical marker were enclosed with a digital pen and then added up. The total stained surface area (addition of all selected areas per square) was divided by the ICT surface area to determine the positive cell proportions (%) of macrophages, M1 and M2 phenotype per sample. To confirm that the stained areas for CD80 and CD206 markers corresponded to macrophage positive cells, every analyzed square was simultaneously observed for CD80 and CD68 or CD206 and CD68 markers. If the stained areas (cells) for M1 or M2 markers did not match with the stained areas for CD68 positive cells, they were not considered for the analysis.

Prior to the analysis, an intra-examiner calibration was performed to determine the consistency and reproducibility of the measurements taken by the described methods. The calibration was accepted when repeated measurements (*n* = 10) presented a intraclass correlation coefficient (ICC) ranging from 0.81 to 1.

### Statistical analysis

The statistical analysis was performed using a commercially available software program (SPSS, 19.0, Chicago, IL, USA). Mean values, standard deviations, and confidence intervals for each variable were calculated at patient level (equal to implant level). The paired *t* test was used to determine statistical significant differences between M1 and M2 expression at slight, moderate, and advanced cases. The one-way ANOVA followed by post hoc Tukey’s test was performed to evaluate significant differences in M1 and M2 expression among groups. The alpha error was set at 0.05. For this analysis, *n* = 6 was considered for each group (i.e., slight, moderate, and advanced). Since the moderate grade group presented *n* = 8 cases, the selection of the 6 samples for inter group analysis was performed through “Simple Random Sampling” in SPSS. Linear regression analyses were performed to depict the relationship between CD68%, M1%, M2%, and M1/M2 ratio and BOP% and PD values. The alpha error was set at 0.05.

Due to the proof-of-principle character of the present study and a lack of similar data in the literature, a sample size calculation was not feasible. However, the initial sample size of *n* = 20 was considered to be sufficient to allow for a first evaluation of the presented histological and clinical data.

## Results

**Fig. 1:**
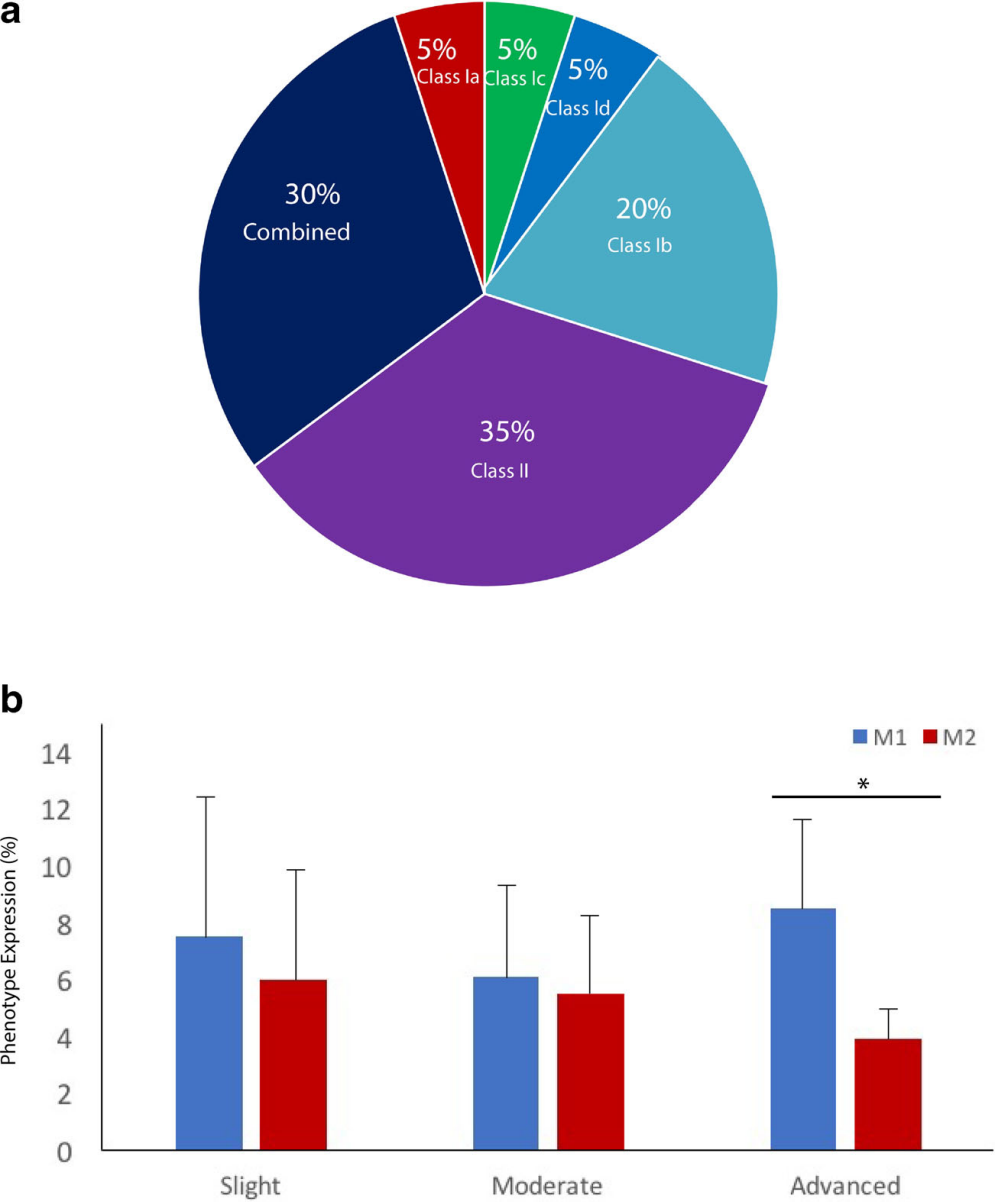
Distribution of **a** defect morphology [22] and **b** bar diagram depicting M1/M2 expression (%) at slight (*n* = 6), moderate (*n* = 6), and advanced (*n* = 6) peri-implantitis cases [19]. *p < 0.05, statistically significant

A total of 20 patients (12 female and 8 male) diagnosed with peri-implantitis who underwent surgical therapy were included in the present analysis. Seven (35%) patients had history of periodontal disease. None of the patients reported smoking habits. The mean implant function was 9.15 ± 6 years before surgical therapy. Implant site characteristics (*n* = 20) and mean values for baseline clinical parameters are summarized in Tables 1 and 2. Peri-implantitis clinical and radiological parameters for slight, moderate, and advanced sites (*n* = 18) are presented in Table 3. The defect morphology distribution is represented in Fig. 1.

**Table 1:** Description of implant site characteristics and frequency distributions

Site characteristic	Number (*n* = 20)	Percentage (%)
Region		
Anterior	3	15
Posterior	17	85
Jaw		
Maxilla	11	55
Mandible	9	45
Bone grafted site		
Yes	7	35
No	13	65
Bone grafting procedure*		
External sinus floor elevation	3	43
Lateral ridge augmentation	4	57
Presence of KM width (< 2 mm)		
Yes	16	80
No (absence)	4	20

*Prior to peri-implantitis surgical therapy

**Table 2:** Clinical parameters (mean, SD, 95% confidence interval (CI) values) for peri-implantitis sites before surgical procedure (*n* = 20)

Clinical parameter	Mean value	SD	95% CI
PI (%)	0.68	0.47	(0.44–0.88)
BOP (%)	71	33	(56.5–85.4)
PD (mm) (probing pocket depth)	5.10	2.16	(4.1–6)
MR (mm)	0.45	1.02	(0.012–0.88)
KM (mm)	3.11	2.07	(2.2–3.9)
MBL (mm)	− 3.63	1.76	(− 4 to − 2.8)
Bone loss (%) (MBL/implant length)	41	19	(32.6–49.6)
Suppuration			
Yes (%)	45	–	
No (%)	55	–	

*PI* plaque index, *BOP* bleeding on probing, *PD* probing depth, *MR* mucosal recession, *KM* keratinized mucosa, *MBL* marginal Bone loss

**Table 3:** Peri-implantitis clinical and radiological parameters for slight, moderate, and advanced sites (*n* = 18)

Peri-implantitis grade	Bone loss (%)	Mean BOP (%)	Mean PD (mm)	Supp (%)
Slight (*n* = 6) 95% CI	16 (12–20)	50 (23–76)	3.6 (2.1–5)	16 ---
Moderate (*n* = 6) 95% CI	36 (33–39)	65 (36–93)	4.9 (3.3–6.5)	50 ---
Advanced (*n* = 6) 95% CI	67 (50–84)	74 (41–106)	5.4 (4.7–5.6)	33.3 ---

**Fig. 2:**
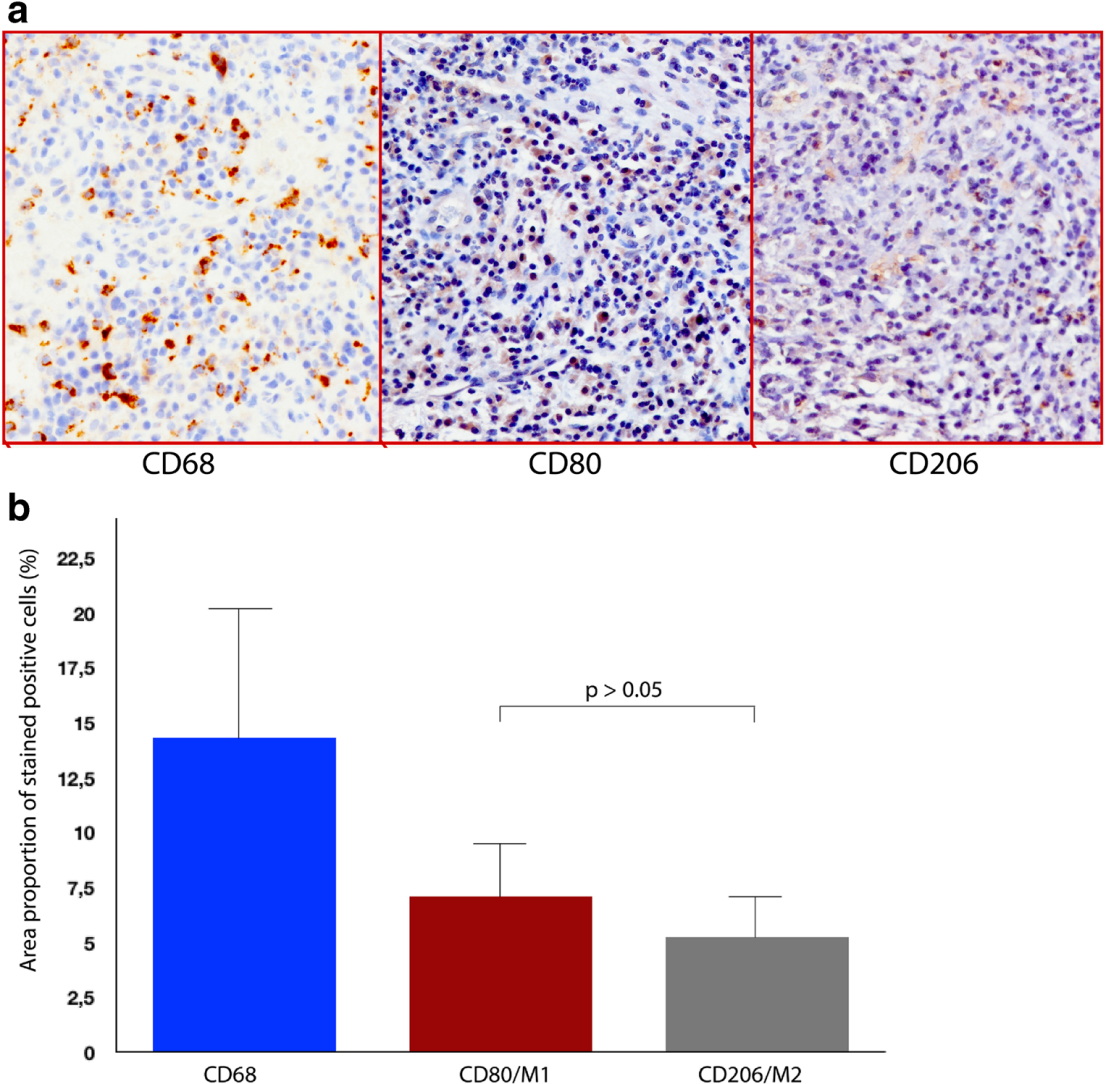
**a** Peri-implantitis histological sections depicting immunohistochemical markers for CD68, CD80, and CD206 (200x). **b** Bar diagram representing the mean proportions (%) expressed per each immunohistochemical marker (i.e., CD68, CD80, and CD206) at the histological sections

The histomorphometrical analysis revealed that the granulation tissue biopsies (sections) had a mean surface area of 7.61 ± 1.4 mm^2^. Respectively, the ICT surface area occupied a total of 2.5 ± 1.9 mm^2^, representing 33% of the biopsy. Micrographs depicting immunohistochemical stainings for CD68, CD80, and CD206 are exemplified in Fig. 2a. Macrophages displaying a positive CD68 expression occupied a mean proportion of 14.36% (95% CI 11.4–17.2) of the ICT area. Positive M1 (CD80) and M2 (CD206) macrophages occupied 7.07% (95% CI 5.9–9.4) and 5.22% (95% CI 3.8–6.6) of the ICT, respectively. There was no significant difference between mean M1 and M2 expression, *p* > 0.05 (Fig. 2b). The mean M1/M2 ratio totaled to 1.56 (95% CI 1.12–1.9).

According to peri-implantitis severity classification [19], slight (*n* = 6), moderate (n = 6), and advanced (n = 6) cases revealed CD68 expression mean values of 13% (95% CI 7–19), 15.8% (95% CI 10.9–20.8), and 12.8% (95% CI 11–14.8), respectively. The latter exhibited mean values for M1 and M2 expression of 7.5% (95% CI 3–12) and 6% (95% CI 2.5–10) (slight grade), 6.1% (95% CI 4–8.2) and 5.5% (95% CI 3.4–7.6) (moderate grade), and 8.5% (95% CI 4.6–12–3) and 3.9% (95% CI 2–5.6) (advanced grade), respectively (Fig. 1). M1% was significantly higher than M2% expression at advanced cases, *p* = 0.01. No significant differences between groups were present when comparing M1 and M2 expression.

The linear regression analysis revealed a significant correlation between CD68 expression (%) and PD values (Coef = 3.18; *R*^2^ = 0.77, *p* = 2.7E-07), showing that higher PD values were significantly associated with a higher expression of macrophages (CD68). As well, a significant correlation between M1 expression (%) and PD values (Coef = 1.65; *R*^2^ = 0.57, *p* = 0.0001) was present, depicting that higher PD values were significantly associated with higher M1% expression. No significant associations between PD, BOP values and M2% expression or M1/M2 ratio were found.

**Fig. 3:**
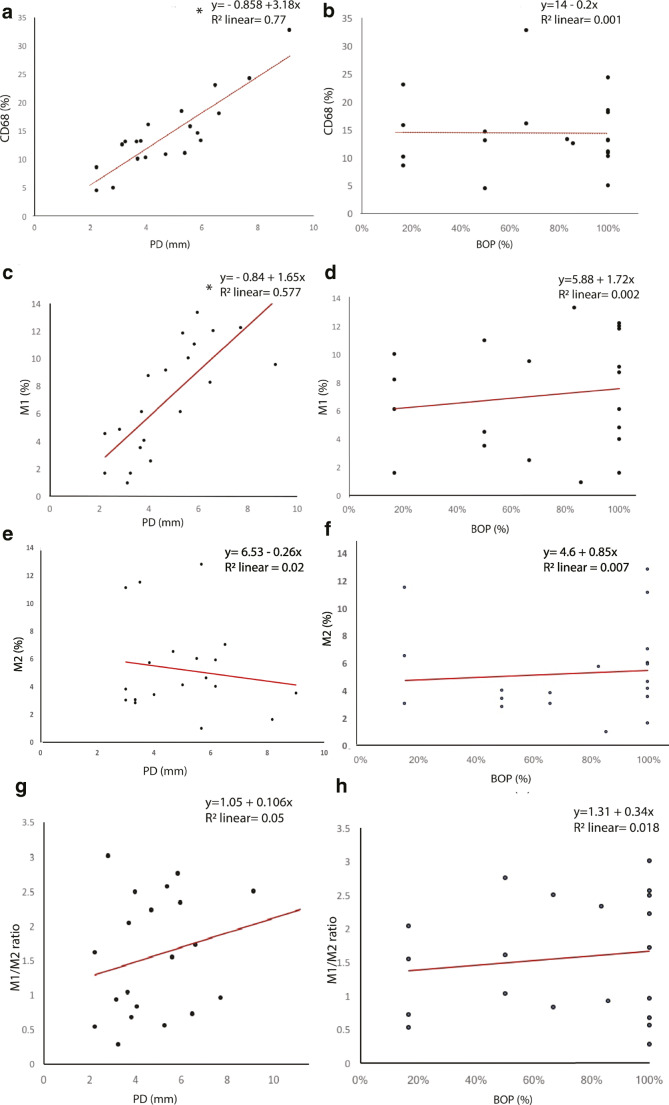
Linear regression plots to represent the relationship between CD68 (%) expression and **a** PD and **b** BOP % values, M1 (%) expression, and **c** PD and **d** BOP % values, M2 (%) expression, and **e** PD and **f** BOP % values and M1/M2 ratio and **g** PD, and **h** BOP % values. *p < 0.05 considered for statistical significance

Linear regression plots depicting the relationship between peri-implantitis clinical parameters (PD and BOP%) and CD68, M1, M2, and M1/M2 ratio are shown in Fig. 3.

## Discussion

The present study aimed at assessing and correlating macrophage polarization M1/M2 status with disease severity at peri-implantitis sites. Within the limitations of the present analysis, the immunohistochemical assessment evidenced that macrophages constitute a distinct proportion of the inflammatory cell infiltrate at peri-implantitis sites, revealing a higher expression for M1 inflammatory phenotype and subsequently an increased M1/M2 ratio, particularly at advanced peri-implantitis cases. The present study also evidenced significant correlations between macrophage and M1 expression (%) and PD values, implying that macrophages might play a considerable role in peri-implantitis onset and progression.

So far, there are limited studies assessing macrophage M1/M2 polarization at human periodontal and peri-implant diseased sites [2, 7, 17]. To authors’ knowledge, this is the first study assessing and associating macrophage polarization with peri-implant disease severity. A recent study evaluating M1/M2 expression in human periodontitis, gingivitis, and healthy biopsies suggested that M1 phenotype was higher in periodontitis tissues, resulting in a higher M1/M2 ratio, and an enhanced expression of inflammatory cytokines [17]. Additionally, Garaicoa–Pazmiño et al. revealed that periodontitis and gingivitis sites contained higher levels of macrophage infiltration when compared with healthy tissues. However, the latter study reported that gingivitis and periodontitis specimens presented a superior M2 expression when compared with the M1 phenotype. Since periodontitis and peri-implantitis exhibit distinct histopathological differences [15], it is not possible to compare the aforesaid results with the present data. Recently, a clinical study compared macrophage polarization distribution at human periodontitis and peri-implantitis lesions at a total of 14 patients (7 biopsies collected per group) and implied that peri-implantitis sites contained a higher macrophage M1 polarization proportion when compared with periodontitis sites [7]. The latter revealed that peri-implantitis samples displayed 14.92 ± 2, 7.06 ± 1.44, and 7.56 ± 1.4% (positive cells) for CD68, M1 (INOS), and M2 (CD206) markers, respectively. These results are closely related with the exposed ones in the present study, particularly for CD68 and M1 expression; however, the present analysis revealed a lower M2 expression. Since different markers have been used to distinguish M1 and M2 phenotypes in the aforementioned studies, a clear limitation exists to compare the reported results. Indeed, the selection of the most accurate molecular markers to determine macrophage M1 and M2 polarization is still a challenging aspect that needs further research [2]. Previous immunohistochemical studies have broadly used CD68 marker to identify macrophages [3, 7, 15], CD80 marker to identify M1 phenotype [23–25] and CD206 to detect M2 phenotype [2, 7, 25]. However, these markers can stain other cells such as fibroblasts, dendritic cells, sub-population of endothelial cells, and B cells [25–27]. Consequently, the present study used CD68 staining as a control to corroborate that positive stained CD206 and CD80 areas corresponded indeed to macrophage expression. Thus, the mentioned methodological limitations should be considered.

Considering that certain pre-clinical and clinical studies have implied that an enhanced M1 inflammatory expression could be related to the pathogenesis of periodontal and peri-implant disease [4, 7, 10, 17], the present study hypothesized that peri-implantitis sites will analogously exhibit a higher M1 macrophage expression. This assumption was supported by the presented results, revealing a higher proportion of the M1 phenotype when compared with the M2 phenotype at peri-implantitis sites, though the reported difference was not statistically significant. Nevertheless, when analyzing and comparing M1/M2 expression at slight, moderate, and advanced cases, a significantly higher M1 expression was exhibited at advanced peri-implantitis sites. In contrast, slight and moderate sites did not reveal any significant difference between M1 and M2 expressions. Additionally, no statistical difference between M1 and M2% was present when comparing the three groups. The mentioned results associating disease severity with macrophage polarization could be related to an immunohistochemical study evaluating macrophage polarization at human soft tissue biopsies retrieved from different disease grades (i.e., healthy, gingivitis, and periodontitis) [17]. This study showed that advanced periodontitis sites had a significantly higher M1 expression when compared with gingivitis and healthy sites. However, no significant difference in M1 expression was found between gingivitis and healthy samples [17]. Furthermore, unexpectedly the latter study showed a significant higher M2 expression at gingivitis samples when compared with healthy ones. Thus, future research is needed for a superior comprehension of the involved immunological mechanisms and role of macrophage polarization in disease progression and severity.

Possibly, a higher expression of M1 phenotype could be related to a “destructive” inflammatory response and a pronounced osteolytic effect around dental implants diagnosed with advanced peri-implantitis [1, 7, 10]. Previous studies have shown that pro-inflammatory reactions at host tissues can be induced by bacteria sub-products (i.e., lipopolysaccharides) or interferon (IFN)-g which subsequently activate M1 phenotype promoting inflammation [10, 28]. In chronic inflammatory diseases, CD4-T-cell-centered processes stimulate T-helper cells to promote or counteract inflammation and osteolysis, playing a pivotal role on macrophage polarization [1, 9, 29]. In this scenario, Th1 and Th17 cells are associated to disease progression, upregulating inflammation, and osteolytic effects. Conversely, Th2 cells and Tregs have been described to reduce disease progression and to decrease inflammation. The Th conversion from Th1 to Th2/Tregs has been related to M2 polarization and the prevention of disease [9, 29]. Conferring to the mentioned immunological processes, several studies have shown that M1 polarization is in fact associated with osteolytic processes [17, 28, 30]. Moreover, some studies have also implied that macrophage switch from M1 to M2 phenotype plays a central role to decrease chronic inflammation and osteolytic effects [9, 28, 30].

Zhou et al. revealed in an immunohistological study that human chronic periodontitis soft tissue samples (*n* = 13) retrieved from diseased sites presenting a mean PD value of 8.23 mm (SD 1.36) had a significant higher M1% expression when compared with gingivitis and healthy samples. This study significantly associated PD values with M1/M2 ratio [17]. Similarly, the present analysis revealed statistically significant correlations between macrophage and M1 (%) expression and PD values. These correlations suggest that a higher macrophage infiltration and M1 inflammatory expression could increase peri-implant pocket depths, disrupting soft tissue adaptation and the “desired” epithelial seal around dental implants. The mentioned disruption could facilitate bacterial invasion into the peri-implant tissue compartments, jeopardizing soft and hard tissue integrity [31]. Still, another limitation of the present study is that the reported M1 and M2 expression at diseased sites could not be compared with healthy peri-implant tissues for obvious ethical reasons. However, this study could elucidate M1 and M2 expression at slight, moderate, and advanced cases, which served as an internal control to compare macrophage polarization at different stages of the disease.

Previous histological studies employing human biopsy material reported that macrophages constitute 5 to 11% of the inflammatory cell composition at peri-implantitis sites [15, 16]. As follows, Berglundh et al. reported in a histopathological analysis of 12 peri-implantitis soft tissue biopsies that macrophages occupied 5.2% of the ICT. In addition, Carcuac and Berglundh evidenced in an immunohistochemical analysis of 40 peri-implantitis soft tissue specimens that positive cells for CD68 marker occupied 11% of the total ICT area (3.48 ± 2.54 mm^2^). The present analysis pointed to a higher macrophage proportion (i.e., 14.36%) at the analyzed peri-implantitis biopsies. Possibly, a superior macrophage expression may be associated with the severity of the diseased sites, which might vary among the studies. However, the discrepancy between the present and previous results could also be attributed to methodological differences between the studies. The aforementioned studies evaluated peri-implantitis inflammatory cell composition at biopsies derived from the supracrestal portion of the diseased site. In contrast, the present analysis had a focus on the evaluation of granulation tissue biopsies which were derived from the intrabony defect component. This was due to the fact that macrophages play a key role in granulation tissue formation [32] and subsequently disease progression. The latter was further investigated by looking at the relationship between CD68%, M1%, M2%, and M1/M2 ratio and BOP% and PD values. Consequently, one of the limitations of the present study is that the peri-implantitis soft tissue component was not analyzed and the total size of the ICT could not be determined.

Within its limitations, the present immunohistochemical study suggests that macrophages reveal a higher expression for M1 inflammatory phenotype and accordingly a higher M1/M2 ratio, particularly at advanced peri-implantitis sites. An enhanced expression of the M1 phenotype could play an important role in peri-implantitis inflammatory response, progression, and osteolytic effect. Subsequently, future research is required to understand the exact mechanisms that lead to tissue destruction and to find possible immune-modulating agents that could modulate M1/M2 status, promoting disease resolution and enhancing tissue repair around dental implants.
